# Supplementary figures and images for: Chronic intake of high-dose of blueberry leaf extract does not augment the harmful effects of ethanol in rats
Source: PeerJ. 2019 Jun 7;7:e6989. doi: 10.7717/peerj.6989 (PMC6557255; doi:10.7717/peerj.6989)

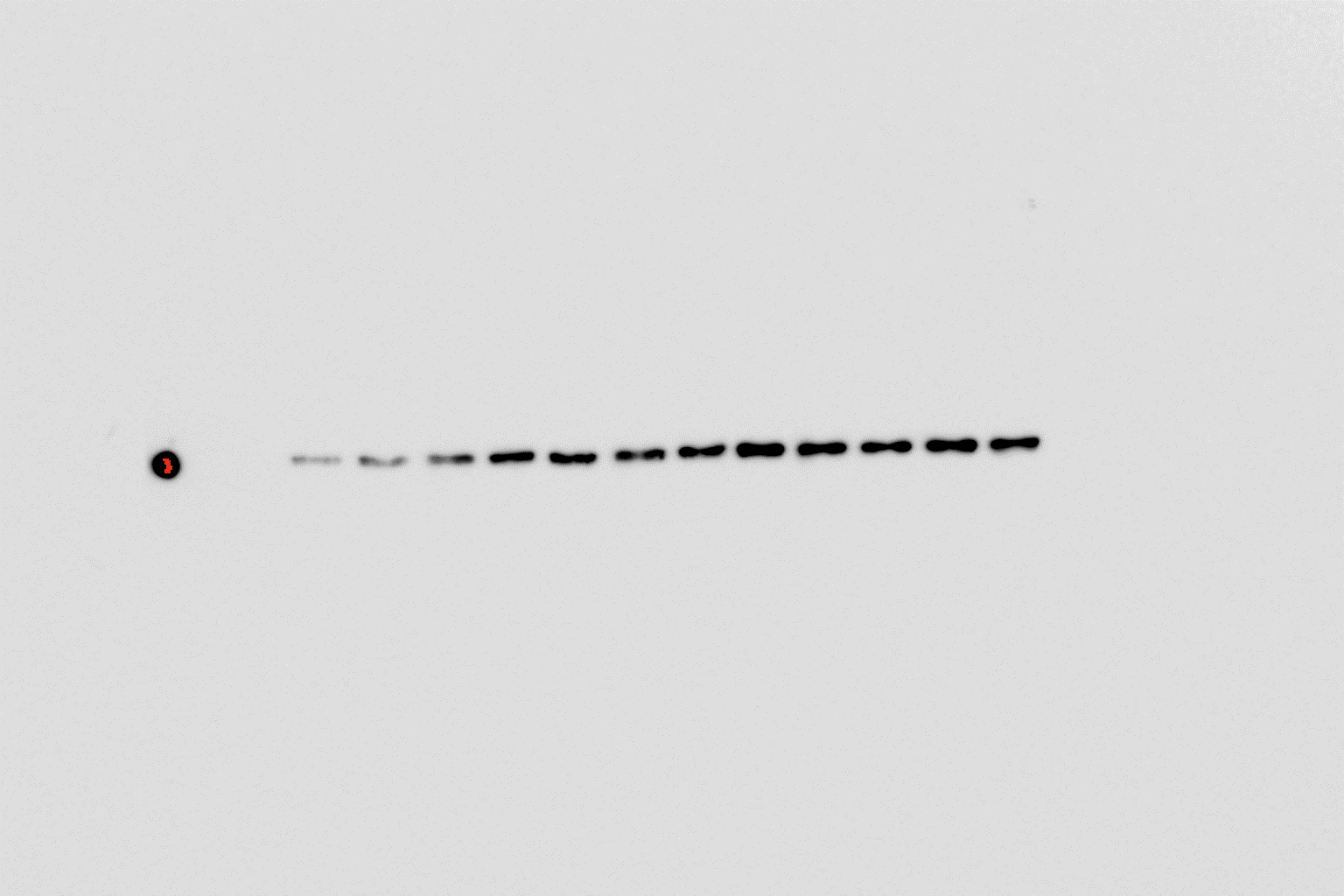

Supplement: Dataset S10 [file peerj-07-6989-s010.zip › CYP2E1-1.png]

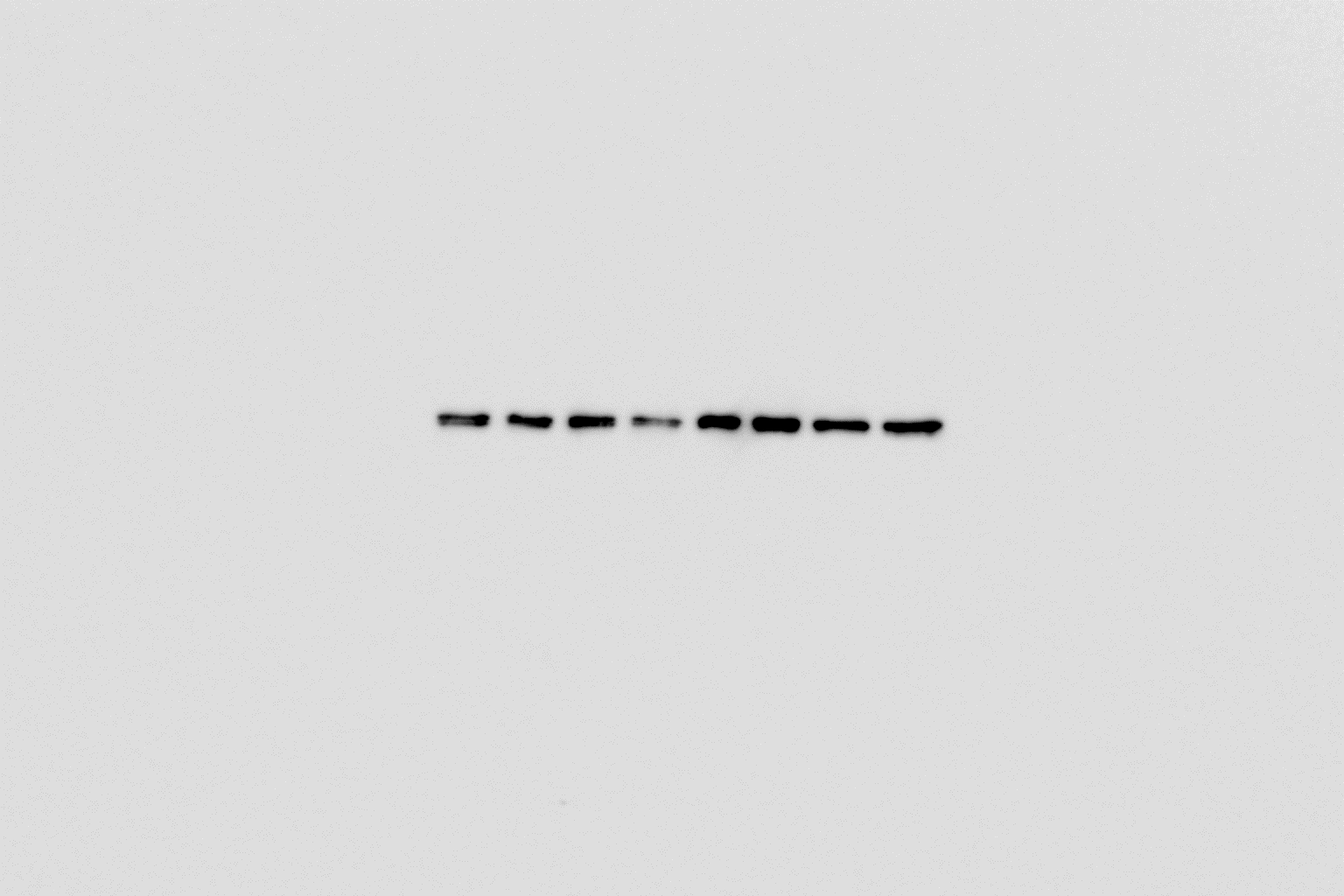

Supplement: Dataset S10 [file peerj-07-6989-s010.zip › CYP2E1-2.png]
